# Prediction of DNA-binding propensity of proteins by the ball-histogram method using automatic template search

**DOI:** 10.1186/1471-2105-13-S10-S3

**Published:** 2012-06-25

**Authors:** Andrea Szabóová, Ondřej Kuželka, Filip Železný, Jakub Tolar

**Affiliations:** 1 Czech Technical University, Department of Cybernetics, Prague, 166 27, Czech Republic; 2 University of Minnesota, Department of Pediatrics, Blood and Marrow Transplantation, Minneapolis, USA

## Abstract

We contribute a novel, *ball-histogram *approach to DNA-binding propensity prediction of proteins. Unlike state-of-the-art methods based on constructing an ad-hoc set of features describing physicochemical properties of the proteins, the ball-histogram technique enables a systematic, Monte-Carlo exploration of the spatial distribution of amino acids complying with automatically selected properties. This exploration yields a model for the prediction of DNA binding propensity. We validate our method in prediction experiments, improving on state-of-the-art accuracies. Moreover, our method also provides interpretable features involving spatial distributions of selected amino acids.

## Introduction

The process of protein-DNA interaction has been an important subject of recent bioinformatics research, however, it has not been completely understood yet. DNA-binding proteins have a vital role in the biological processing of genetic information like DNA transcription, replication, maintenance and the regulation of gene expression. Several computational approaches have recently been proposed for the prediction of DNA-binding function from protein structure.

In the early 80's, when the first three-dimensional structures of protein-DNA complexes were studied, Ohlendorf and Matthew [1] noticed that the formation of protein-DNA complexes is energetically driven by the electrostatic interaction of asymmetrically distributed charges on the surface of the proteins complementing the charges on DNA. Large regions of positive electrostatic potentials on protein surfaces have been suggested to be a good indication of DNA-binding sites.

Stawiski et al. [2] proposed a methodology for predicting Nucleic Acid-binding function based on the quantitative analysis of structural, sequence and evolutionary properties of positively charged electrostatic surfaces. After defining the electrostatic patches they found the following features for discriminating the DNA-binding proteins from other proteins: secondary structure content, surface area, hydrogen-bonding potential, surface concavity, amino acid frequency and composition and sequence conservation. They used 12 parameters to train a neural network to predict the DNA-binding propensity of proteins.

Jones et al. [3] analysed residue patches on the surface of DNA-binding proteins and developed a method of predicting DNA-binding sites using a single feature of these surface patches. Surface patches and the DNA-binding sites were analysed for accessibility, electrostatic potential, residue propensity, hydrophobicity and residue conservation. They observed that the DNA-binding sites were amongst the top 10% of patches with the largest positive electrostatic scores.

Tsuchiya et al. [4] analysed protein-DNA complexes by focusing on the shape of the molecular surface of the protein and DNA, along with the electrostatic potential on the surface, and constructed a statistical evaluation function to make predictions of DNA interaction sites on protein molecular surfaces.

Ahmad and Sarai [5] trained a neural network based on the net charge and the electric dipole and quadrupole moments of the protein. It was found that the magnitudes of the moments of electric charge distribution in DNA-binding protein chains differ significantly from those of a non-binding control data set. It became apparent that the positively charged residues are often clustered near the DNA and that the negatively charged residues either form negatively charged clusters away from the DNA or get scattered throughout the rest of the protein. The entire protein has a net dipole moment, because of the topological distribution of charges. The resulting electrostatic force may steer proteins into an orientation favorable for binding by ensuring that correct side of the protein is facing DNA.

Bhardwaj et al. [6] examined the sizes of positively charged patches on the surface of DNA-binding proteins. They trained a support vector machine classifier using positive potential surface patches, the protein's overall charge and its overall and surface amino acid composition. In case of overall composition, noticeable differences were observed between the binding and the non-binding case with respect to the frequency of Lys and Arg. These are positively charged amino acids, so their over-representation in DNA-binding proteins is evident.

A further advancement in DNA binding propensity prediction was presented by Szilágyi and Skolnick [7]. Their method was based on a logistic regression classifier with ten variables (physicochemical properties) to predict from sequence and low-resolution structure of a protein whether it is DNA-binding. To find features that discriminate between DNA-binding and non-DNA-binding proteins, they tested a number of properties. The best combination of parameters resulted in the amino acid composition, the asymmetry of the spatial distribution of specific residues and the dipole moment of the protein.

The above approaches rely exclusively on protein structure data (whether sequential or spatial). To our knowledge, the predictive accuracy achieved by the lastly mentioned strategy [7] was only improved by incorporating an additional source of background knowledge, in particular, information on evolutionarily conserved domains. Nimrod et al. [8] presented a random forest classifier for identifying DNA-binding proteins among proteins with known 3D structures. First, their method detects clusters of evolutionarily conserved regions on the surface of proteins using the PatchFinder algorithm. Next, a classifier is trained using features like the electrostatic potential, cluster-based amino acid conservation patterns, the secondary structure content of the patches and features of the whole protein, including all the features used by Szilágyi and Skolnick [7].

It is nevertheless important to continue improving methods that do not exploit evolutionary information. Such methods are valuable mainly due to their ability to predict DNA-binding propensity for engineered proteins for which evolutionary information is not available. Engineered proteins are highly significant for example in emerging gene-therapy technologies [9].

In this paper we will be concerned with prediction of DNA-binding propensity from spatial structure information without using evolutionary information. To this end, we propose the *ball-histogram *method, which improves on the state-of-the-art approaches in the following way. Rather than constructing an ad-hoc set of features describing the physicochemical properties of the proteins, we base our approach on a systematic, Monte-Carlo-style exploration of the spatial distribution of amino-acids complying to automatically selected properties. For this purpose we employ so-called ball histograms, which are capable of capturing joint probabilities of these specified amino acids occurring in certain distances from each other. Another positive aspect of our method is that it provides us with interpretable features involving spatial distributions of selected amino acids.

## Data

DNA-binding proteins are proteins containing DNA-binding domains. A DNA-binding domain is an independently folded protein domain that contains at least one motif that recognizes double- or single-stranded DNA. We investigate structural relations within these proteins following the spatial distributions of certain amino acids in available DNA-protein complexes.

**Figure 1:**
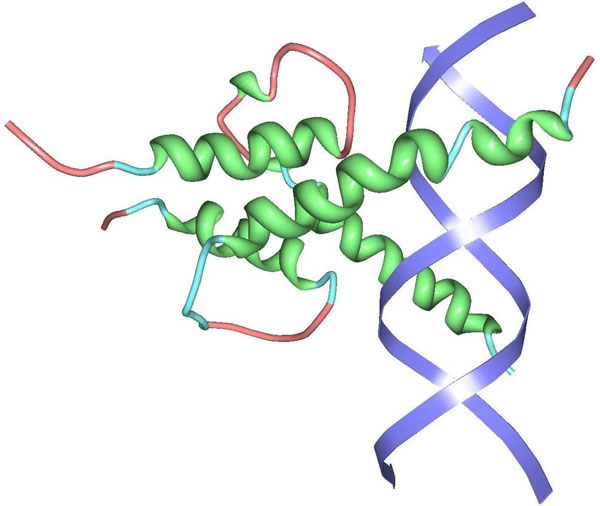
**Exemplary DNA-binding protein in complex with DNA**. Exemplary DNA-binding protein in complex with DNA shown using the protein viewer software [22]. Secondary structure motifs are shown in green (*α*-helices), light blue (turns) and pink (coils); the two DNA strands are shown in blue.

We decided to work with a positive data set (PD138) of 138 DNA-binding protein sequences in complex with DNA. It was created using the Nucleic Acid Database by [7] - it contains a set of DNA-binding proteins in complex with DNA strands with a maximum pairwise sequence identity of 35% between any two sequences. Protein structures have ≤ 3.0Å resolution. An example DNA-binding protein in complex with DNA is shown in Figure 1.

Rost and Sander constructed a dataset (RS126) for secondary structure prediction. Ahmad & Sarai [5] removed the proteins related to DNA binding from it, thus getting a final dataset of non-DNA-binding proteins. We used this set of non-DNA-binding proteins as our negative dataset (NB110).

We also used an extended dataset (NB843) by Nimrod et al. [8]. This dataset contains additional 733 structures of non-DNA-binding proteins. The additional structures were gathered using the PISCES server.

Entries in this list include crystal structures with a resolution better than 3.0Å. The sequence identity between each pair of sequences is smaller than 25%.

From the structural description of each protein we extracted the list of all contained amino acids with information on their type and spatial structure.

## Method

In this section we describe our novel method for predictive classification of DNA-binding propensity of proteins using so-called *ball histograms*. The motivation for the method is the observation that distributions of certain types of amino acids differ significantly between DNA-binding and non-DNA-binding proteins. This suggests that information about distributions of some amino acids could be used to construct predictive models able to classify proteins as binding or non-binding given their spatial structure. We propose the following approach which is able to capture fine differences between the distributions. It consists of four main parts. First, so-called *templates *are found, which determine amino acids whose distributions should be captured by *ball histograms*. In the second step *ball histograms *are constructed for all proteins in a training set. Third, a transformation method is used to convert these histograms to a form usable by standard machine learning algorithms. Finally, a random forest classifier [10] is learned on this transformed dataset and then it is used for classification. The reason why we chose the random forest learning algorithm is that it is known to be able to cope with large numbers of attributes such as in our case of *ball histograms *[11]. We also experimented with the Support Vector Machine method in the third step, however, it was consistently outperformed by the random forest classifier.

### Ball histograms

A *template *is a list of some Boolean amino acid properties. A property may, for example, refer to the charge of the amino acid (e.g. *Positive*), but it may also directly stipulate the amino acid type (e.g. *Arginine*). An example of a template is (*Arg, Lys, Polar*) or (*Positive, Negative, Neutral*). A *bounding sphere *of a protein structure is a sphere with center located in the geometric center of the protein structure and with radius equal to the distance from the center to the farthest amino acid of the protein plus the diameter of the *sampling ball *which is a parameter of the method. We say that an amino acid *falls *within a sampling ball if the alpha-carbon of that amino acid is contained in the sampling ball in the geometric sense.

Given a protein structure, a template *τ *= (*f*_1_, ..., *f_k_*), a sampling-ball radius *R *and a bounding sphere *S*, a *ball histogram *is defined as:

(1) $$H_{\tau }\left(t_{1},\;\ldots ,\;t_{k}\right)=\frac{{\int \int \int }_{\left(x,y,z\right)\in S}I_{T,R}\left(x,y,z,t_{1},\ldots ,t_{k}\right)dxdydz}{\sum_{\left(t_{1}^{\prime },..,t_{k}^{\prime }\right)}{\int \int \int }_{\left(x,y,z\right)\in S}I_{T,R}\left(x,y,z,t_{1}^{\prime },\ldots ,t_{k}^{\prime }\right)dxdydz}$$

where *I_T, R_*(*x, y, z, t*_1_, ..., *t_k_*) is an indicator function which we will define in turn. The expression $\sum_{\left(t_{1}^{\prime },\ldots ,t_{k}^{\prime }\right)}{\int \int \int }_{\left(x,y,z\right)\in S}I_{T,R}\left(x,y,z,t_{1}^{\prime },\;\ldots ,\;t_{k}^{\prime }\right)dxdydz$ is meant as a normalization factor - it ensures that $\sum_{\left(t_{1},\ldots ,t_{k}\right)}H_{T}\left(t_{1},\ldots ,t_{k}\right)=1$. In order to define the indicator function *I*_*T*_, _*R *_we first need to define an auxiliary indicator function $I_{T,R}^{\prime }\left(x,y,z,t_{1},\;\ldots ,\;t_{k}\right)$

$$I_{T,R}^{\prime }\left(x,\;y,\;z,\;t_{1},\;\ldots ,\;t_{k}\right)=\left\{\begin{array}{cc} 1 & \begin{array}{c} \text{if there are exactly }t_{i}\text{ amino acids complying with property }f_{i}\\ \left(\text{1 }\leq i\;\leq k\right)\text{ in the sampling ball with center }x,\;y,\;z\text{ and radius }R, \end{array}\\ 0 & \text{otherwise}\text{.} \end{array}\right)$$

Notice that $I_{T,R}^{\prime }\left(x,y,z,0,\ldots ,0\right)$ does not make any distinction between a sampling ball that contains no amino acid at all and a sampling ball that contains some amino acids of which none complies with the parameters in the template *T*. Therefore if we used $I_{T,R}^{\prime }$ in place of *I_T, R _*the histograms would be affected by the amount of empty space in the bounding spheres. Thus, for example, there might be a big difference between histograms of otherwise similar proteins where one would be oblong and the other one would be more curved. In order to get rid of this unwanted dependence of the indicator function *I_T;R _*on proportion of empty space in sampling spheres we define *I_T, R _*in such a way that it ignores the empty space. For (*t*_1_,..., *t_k_*) ≠ 0 we set

$$I_{T,R}\left(x,\;y,\;z,\;t_{1},\;\ldots ,\;t_{k}\right)=I_{T,R}^{\prime }\left(x,\;y,\;z,\;t_{1},\;\ldots ,\;t_{k}\right).$$

In the cases when (*t*_1_, ..., *t_k_*) = 0 we set *I_T, R_*(*x, y, z, t*_1_, ..., *t_k_*) = 1 if and only if $I_{T,R}^{\prime }\left(x,y,z,t_{1},\ldots ,t_{k}\right)=1$ and if the sampling ball with radius *R *at (*x, y, z*) contains at least one amino acid.

**Figure 2:**
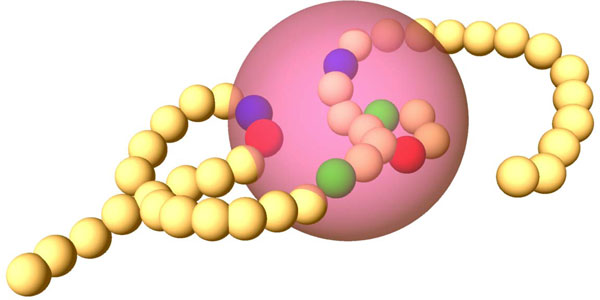
**Illustration of the Ball Histogram Method**. Amino acids are shown as small balls in sequence forming an amino acid chain. A *sampling ball *is shown in violet. Some of the amino acids which comply with properties of an example template are highlighted inside the *sampling ball *area. They have different colors according to their type.

Ball histograms capture the joint probability that a randomly picked *sampling ball *(See Figure 2) containing at least one amino acid will contain exactly *t*_1 _amino acids complying with property *f*_1_, *t*_2 _amino acids complying with property *f*_2 _etc. They are invariant to rotation and translation of protein structures which is an important property for classification. Also note that the histograms would not change if we increased the size of the bounding sphere.

The indicator function *I_T, R _*makes crisp distinction between the case where an amino acid falls within a sampling ball on one hand, and the case where it falls out of it, on the other hand. This could be changed towards capturing a more complex case by replacing the value 1 by the fraction of the amino acid that falls within the sampling ball, however, for simplicity we will not consider this case in this paper.

### Ball-histogram construction

Computing the integral in Equation 1 precisely is infeasible therefore we decided to use a Monte-Carlo method. The method starts by finding the bounding sphere. First, the geometric center *C *of all amino acids of a given protein *P *is computed (each amino acid is represented by coordinates of its alpha-carbon). The radius *R_S _*of the sampling sphere for the protein structure *P *is then computed as

$$R_{S}=\underset{Res\in P}{\text{max}}\left(distance\left(Res,\;C\right)\right)+R$$

where *R *is a given sampling-ball radius. After that the method collects a pre-defined number of samples from the bounding sphere. For each sampling ball the algorithm counts the number of amino acids in it, which comply with the particular properties contained in a given template and increments a corresponding bin in the histogram. In the end, the histogram is normalized.

**Figure 3:**
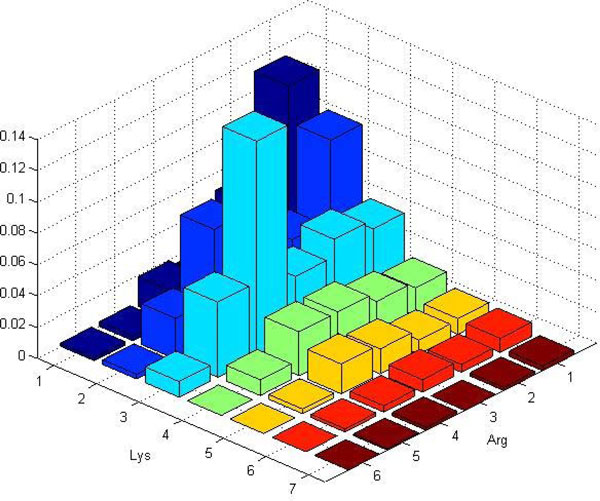
**Example ball histogram constructed for protein 1A31**. Example ball histogram with template (*Arg, Lys*) and sampling-ball radius *R *= 12 Åconstructed for proteins 1A31 from PD138.

**Figure 4:**
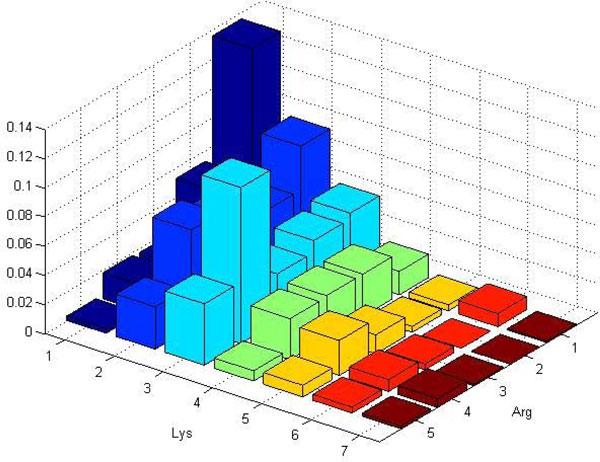
**Example ball histogram constructed for protein 1A3Q**. Example ball histogram with template (*Arg, Lys*) and sampling-ball radius *R *= 12 Åconstructed for proteins 1A3Q from PD138.

**Example 1**. *Let us illustrate the process of histogram construction. Consider the template *(*Arg, Lys*) *and assume we already have a bounding sphere. The algorithm starts by placing a sampling ball randomly inside the bounding sphere. Assume the first such sampling ball contained the following amino acids: *2 Arginins and 1 Leucine *therefore we increment (by 1) the histogram's bin associated with vector *(2, 0). *Then, in the second sampling ball, we get *1 Histidine and 1 Aspartic acid, *so we increment the bin associated with vector *(0, 0). *We continue in this process until we have gathered a sufficient number of samples. In the end we normalize the histogram. Examples of such histograms are shown in *Figure 3 and 4.

### Predictive classification using ball histograms

In the preceding sections we have explained how to construct ball-histograms but we have not explained how we can use them for predictive classification. One possible approach would be to define a metric on the space of normalized histograms and then use either a nearest neighbour classifier or a nearest-centroid classifier. Since our preliminary experiments with these classifiers did not give us satisfying predictive accuracies, we decided to follow a different approach inspired by a method from relational learning known as *propositionalization *[12] which is a method for transferring complicated relational descriptions to attribute-value representations.

The transformation method is quite straightforward. It looks at all histograms generated from the protein structures in a training set and creates a numerical *attribute *for each vector of property occurrences which is non-zero at least in one of the histograms. After that an attribute vector is created for each training example using the collected attributes. The values of the entries of the attribute-vectors then correspond to heights of the bins in the respective histograms. After this transformation a random forest classifier is learned on the attribute-value representation. This random forest classifier is then used for the predictive classification.

In practice, there is a need to estimate the optimal sampling-ball radius. This can be done by creating several sets of histograms and their respective attribute-value representations corresponding to different radii and then selecting the optimal parameters using an internal cross-validation procedure.

### Construction of templates

A question that we left unanswered so far in the description of our method is how to construct appropriate templates, which would allow us to accurately predict DNA-binding propensity. It is obvious that an *all-inclusive *strategy where the template would simply list all possible properties is infeasible. A template with *n *properties will generate training samples with a number of attributes *d *that is exponential in *n*. Furthermore, machine learning theory [13] indicates that the number of training samples needed to preserve accurate classification grows exponentially in *d*. In effect, the requested number of training samples grows doubly-exponentially with the size of the template. It is thus crucial that the template consists of only a small number of relevant properties. On the other hand, omitting some amino acids completely might be a problem as well. A possible solution is to use more templates of bounded size instead of one big template, because the number of attributes *d *grows only linearly with the number of templates.

One possibility could be to use templates with properties corresponding to amino acid types believed to play an important role in the DNA-binding process according to literature. This could mean, for example, using the four charged amino acids as properties in a template - *Arg, Lys, Asp, Glu*, which are known to often interact with the negatively charged backbone as well as with the bases of the DNA [14-16] or other amino acids identified as important, e.g. the eight amino acids used in [7]. We performed such experiments in [17].

Here we advance the strategy by developing an automated method for template construction. The basic idea of the method is to find templates which maximize *distance *between average histograms from the two classes (DNA-binding and non-DNA-binding proteins). Intuitively, such templates should allow us to construct classifiers with good discriminative ability. We construct the templates in a heuristic way using a variant of best-first search algorithm (Algorithm 1 - Template Search) to maximize the distance between the average histograms from the two classes. In this paper we use the Bhattacharyya distance [18] which is defined as

$$D_{B}\left(h_{a},\;h_{b}\right)=-\text{ln}\left(\underset{x\in X}{\sum }\sqrt{h_{a}\left(x\right)\cdot h_{b}\left(x\right)}\right),$$

where *h_a _*and *h_b _*are histograms and *X *is their support-set. Further different *distances *could be used as well.

The following example shows that the templates cannot be constructed greedily. Although we do not directly prove hardness of the *template-search *problem here, the next example gives an intuition why the problem is probably hard.

**Example 2**. *We assume that we have histograms for DNA-binding proteins and non-DNA-binding proteins as shown below and we want to find an optimal template with length 2. It can be easily verified that greedy search starting with an empty template would construct either the template *(Arg, Gly) *or *(Lys, Gly), *but not the optimal template *(Arg, Lys).

**Table 3:** DNA-binding proteins:

**Lys**				**Gly**				**Gly**			
					
**Arg**	**0.5**	**0**	0.5	**Arg**	**0.4**	**0.1**	0.5	**Lys**	**0.4**	**0.1**	0.5
	**0**	**0.5**	0.5		**0.2**	**0.3**	0.5		**0.2**	**0.3**	0.5
					
	0.5	0.5			0.6	0.4			0.6	0.4	

**Table 4:** Non-DNA-binding proteins:

**Lys**				**Gly**				**Gly**			
					
**Arg**	**0**	**0.5**	0.5	**Arg**	**0.1**	**0.4**	0.5	**Lys**	**0.1**	**0.4**	0.5
	**0.5**	**0**	0.5		**0.3**	**0.2**	0.5		**0.3**	**0.2**	0.5
					
	0.5	0.5			0.4	0.6			0.4	0.6	

*Consider now the case where the search algorithm starts from the empty template. In the first step, the template *(Gly) *is constructed because it maximizes distance between the histograms for the two classes*.

*Both *(Arg) *and *(Lys) *would give rise to identical histograms for the two classes. In the next step*, Arg *or *Lys *is added to this template. However, the resulting template is clearly not optimal as can be checked by routine calculation of the Bhattacharyya distance which is finite for the discovered sub-optimal template but which would be infinite for the optimal template *(Arg, Lys).

*Similarly, if we wanted to construct an optimal template of length 1 and if we started with the maximal template *(Arg, Lys, Gly) *and then tried to iteratively remove its elements while greedily maximizing the distance between the histograms of the two classes then we would end up with a sub-optimal template as before. In the first step*, Gly *would be removed and we would get the optimal template of size 2 *(Arg, Lys). *However, in this case we want to construct a template of length 1. Therefore, in the next step we would create either the template *(Arg) *or *(Lys) *but not the optimal one *(Gly) *(note that the distances for templates *(Arg) *or *(Lys) *are 0*).

In order to avoid repeated construction of histograms from the whole datasets, we construct a histogram corresponding to the largest possible template (containing all amino acid properties), then, during the best-first search, we construct histograms for the other templates by marginalising this largest histogram. While searching for a single template using best-first search is quite straightforward, searching for several templates is more complicated. This is because we need to find not only a set of templates making the distances between the average histograms as large as possible, but also these templates should be sufficiently diverse. There are several possible ways to enforce diversity in the template set, and we decided to follow a fast heuristic approach. During the template search we penalize all candidate templates which are subsets of some templates already discovered in previous runs of the procedure.

**Algorithm 1 **Template Search

**function ***TemplateSearch*()

*Templates ← *{}

**for ***i *= 1 to *NumberO fTemplates ***do**

*i *← *i *+ 1

*Templates ← Templates *∪ *BestFirstSearch*(*Templates*)

end for

**function ***BestFirstSearch*(*Templates*, λ)

*E*^+ ^- set of positive examples (DNA-binding Proteins)

*E^- ^*- set of negative examples (non-DNA-binding Proteins)

*Open ← *{()}

*Open ← Open *∪ {*t_i _∩ t_j _|t_i_, t_j _Templates*}

*Closed ←*∅

*BestTemplate ← *()

*Scores ← HeuristicScore*(*Open*)

**while ***Open ≠* ∅ **do**

*Template ← *Remove best template from *Open *according to *Scores*

**if **(*D_x_*(*Template, E*^+^, *E^-^*) *> D_x_*(*BestTemplate, E*^+^, *E^-^*)) ∧ (*Template *∉ *Templates*) **then**

BestTemplate ← Template

end if

**for ***T *∈ *Expand*(*Template*) **do**

**if ***T *∉ *Closed ***then**

*Closed ← Closed *∪ {*T*}

*Open ← Open *∪ {*T*}

**if **∃ *T^' ^*∈ *Templates*: *T *⊆ *T^' ^***then**

*Score ← *λ · *HeuristicScore*(*T*)

else

*Score ← HeuristicScore*(*T*)

end if

*Scores ← Scores *∪ *Score*

end if

end for

end while

*BestFirstSearch(Templates)*. In order to direct the search early to the most promising regions of the search-space, we first initialize the set *Open *with all pairwise intersections of the already discovered templates. Intuitively, sub-templates which appear in more templates constitute a kind of a *core *shared by the most informative templates. This, together with penalization of *redundant *templates, helps the algorithm visit the most promising parts of the search space.

## Results

In this section we present experiments performed on real-life data described in section Data. We constructed histograms with automatically discovered templates and three different sampling-ball radii: 4, 8 and 12 Å. We trained random forest classifiers selecting optimal sampling-ball radii and optimal numbers of templates (1, 3, 5 or 7 templates) for each fold by internal cross-validation. The estimated AUCs (area under curve) are shown in Table 1 and the estimated accuracies are shown in Table 2. We performed two sets of experiments. In the first experiment we tested the ball histogram method with settings as described above (see *Ball Histograms *in Tables 1 and 2). In the second experiment we used only the coarse-grained features of [7]. For both of these experiments we trained two types of classifiers: random forests [10] and linear support vector machines [19] in order to determine the extent to which the choice of classifiers matters. In addition, we learnt a logistic regression classifier [20] for the experiment with the coarse-grained features from [7] since it was the classifier used originally by the authors of [7]. (We also tried to learn logistic regression classifiers for the ball-histogram method but logistic regression turned out to be too slow with the high number of attributes generated by the ball-histogram method.) We can see from the experimental results that the choice of the classifier has low influence on the performance of the Szilágyi's and Skolnick's method whereas it has slightly bigger impact on the ball-histogram method. A possible explanation is that random forest classifier is able to cope with large numbers of attributes [11] such as in our ball histogram method.

**Table 1:** AUCs estimated by 10-fold cross-validation.

	Classifier	PD138/NB110	PD138/NB843
Ball Histograms	Random Forest	**0.94 **± **0.05**	**0.87 **± **0.04**
	SVM	0.92 ± 0.04	0.83 ± 0.03

	Logistic Regression	0.92 ± 0.04	0.84 ± 0.04
Szilágyi and Skolnick [7]	Random Forest	0.90 ± 0.05	0.82 ± 0.04
	SVM	0.92 ± 0.05	0.83 ± 0.05

**Table 2:** Accuracies estimated by 10-fold cross-validation.

	Classifier	PD138/NB110	PD138/NB843
Ball Histograms	Random Forest	**0.87 **± **0.08**	**0.88 **± **0.01**
	SVM	0.84 ± 0.07	0.87 ± 0.01

	Logistic Regression	0.81 ± 0.05	0.87 ± 0.01
Szilágyi and Skolnick [7]	Random Forest	0.82 ± 0.07	0.87 ± 0.02
	SVM	0.81 ± 0.05	0.87 ± 0.01

We report both AUC and accuracy for the two combinations of datasets (PD138/NB110 and PD138/NB843). In case of datasets PD138 and NB110, the ball-histogram method achieved the best results in terms of accuracy and AUC. The best results for ball-histogram methods were obtained by random forest learning algorithm. In case of datasets PD138 and NB843 accuracy is not very meaningful measure of classification quality because the dataset is highly class-skewed. However, if we have a look at the AUC value, we can see that again the ball-histogram method performs best.

In order to see whether the ball-histogram method, which uses only structural information, could come close to the results of methods which exploit also information about the evolutionary conservation of regions on protein surfaces, we compared our results with the results of Nimrod et al. [8]. The AUC 0.96 and accuracy 0.90 reported in [8] for the datasets PD138 and NB110 differs only slightly (0.02, 0.03 respectively) from our best results. The AUC 0.90 obtained for the datasets PD138 and NB843 differs by 0.03 from our best results. These results are encouraging given how important evolutionary information turned out to be according to experiments from [8]. When removing evolutionary information, their classifier's misclassification error increased by 0.035. Even without this information their classifier used significantly more information than our method (e.g. secondary structure information).

In addition to improved accuracy, our method provides us with interpretable features involving distributions of selected amino acids in protein structures. The three most informative automatically selected templates are: *(Arg, Cys, Gly*), *(Cys, Gly, Positive*), *(Cys, Polar, Positive*). It is well-known that positively charged amino acids (under normal circumstances *Arg *and *Lys *are positively charged, *Glu *and *Asp *are charged negatively) are critical for DNA-binding function ([14-16]). This is probably the reason why the property *Positive *is contained in two out of three most informative templates. In the third one we have the explicitly listed positively charged amino acid - *Arginine*. The remaining amino acid properties listed in the three most informative templates also fit well with the results of Sathyapriya et al. [21], where protein-DNA interactions were studied through structure network analysis. According to their results the polar, non-negative amino acids have high DNA-binding propensity which supports meaningfulness of the template *(Cys, Polar, Positive*). Furthermore, they also show that *Cysteine *is one of the amino acids with the lowest DNA-binding propensity. This again fits well with the discovered templates, since *Cysteine *appears in all of them.

**Figure 5:**
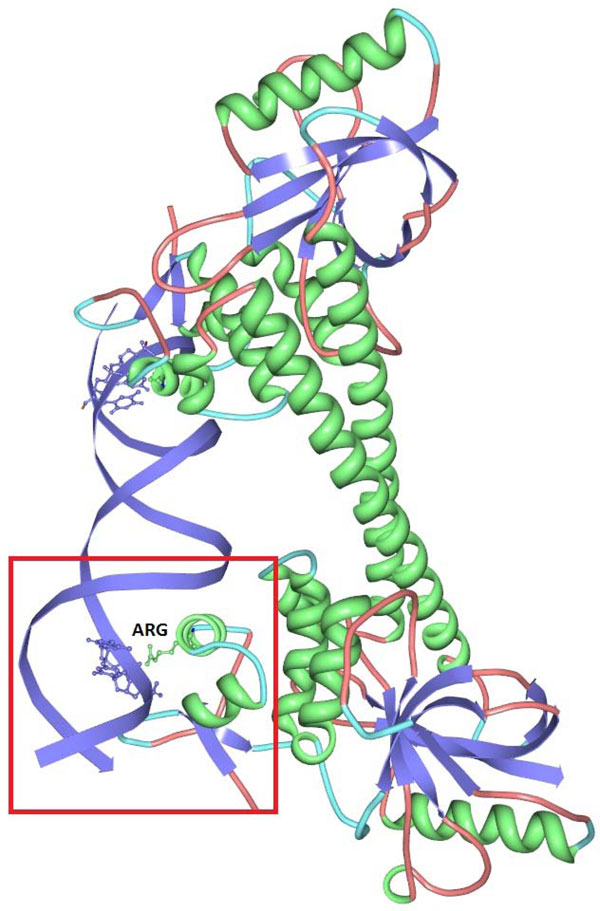
**Discovered pattern example**. Protein 1R8E containing the discovered pattern (*Arg, Cys, Gly*) = (1, 0, 0) shown using the protein viewer software [22]. Amino acid assumed by the pattern is indicated.

Each template gives rise to a set of features which correspond to individual bins in the respective multi-dimensional histogram. It is therefore interesting to evaluate also the particular features from the point of view of predictive information which they carry. We evaluated the features corresponding to the automatically selected templates using *χ*^2^-criterion. The most informative *feature *according to the *χ*^2^-criterion assumed presence of one arginine, no cysteine and no glycine in a ball with radius 8 Å. Given a protein structure, each feature captures the fraction of sampling balls, which contain the specified numbers of amino acids complying with given properties. The next two most informative features assumed presence of two, respectively three positively charged amino acids, no cysteine and no glycine. We show an example occurrence of the first feature in a DNA-binding protein with the highlighted amino acid in Figure 5. All the three most informative features correspond to the above-mentioned observations from [21].

## Conclusions

We improved on state-of-the-art accuracies in the prediction of DNA-binding propensity of proteins from structure data through an innovative *ball histogram method*. The method is based on systematic exploration of the distribution of automatically-selected amino acid properties in protein structures, yielding a predictive model based on features amenable to direct interpretation.

## Competing interests

The authors declare that they have no competing interests.

## Authors' contributions

AS and OK conceived, designed and implemented the methods, performed the experiments and analysed the results. AS, OK, FŽ and JT wrote the paper. All authors read and approved the manuscript.
